# A Novel Autophagy-Related Prognostic Risk Model and a Nomogram for Survival Prediction of Oral Cancer Patients

**DOI:** 10.1155/2022/2067540

**Published:** 2022-01-06

**Authors:** Hongjun Fei, Xiongming Chen

**Affiliations:** Department of Reproductive Genetics, International Peace Maternity and Child Health Hospital, Shanghai Key Laboratory of Embryo Original Diseases, Shanghai Municipal Key Clinical Specialty, Shanghai Jiao Tong University School of Medicine, Shanghai 200030, China

## Abstract

**Background:**

This study is aimed at constructing a risk signature to predict survival outcomes of ORCA patients.

**Methods:**

We identified differentially expressed autophagy-related genes (DEARGs) based on the RNA sequencing data in the TCGA database; then, four independent survival-related ARGs were identified to construct an autophagy-associated signature for survival prediction of ORCA patients. The validity and robustness of the prognostic model were validated by clinicopathological data and survival data. Subsequently, four independent prognostic DEARGs that composed the model were evaluated individually.

**Results:**

The expressions of 232 autophagy-related genes (ARGs) in 127 ORCA and 13 control tissues were compared, and 36 DEARGs were filtered out. We performed functional enrichment analysis and constructed protein–protein interaction network for 36 DEARGs. Univariate and multivariate Cox regression analyses were adopted for searching prognostic ARGs, and an autophagy-associated signature for ORCA patients was constructed. Eventually, 4 desirable independent survival-related ARGs (*WDR45*, *MAPK9*, *VEGFA*, and *ATIC*) were confirmed and comprised the prognostic model. We made use of multiple ways to verify the accuracy of the novel autophagy-related signature for survival evaluation, such as receiver-operator characteristic curve, Kaplan–Meier plotter, and clinicopathological correlational analyses. Four independent prognostic DEARGs that formed the model were also associated with the prognosis of ORCA patients.

**Conclusions:**

The autophagy-related risk model can evaluate OS for ORCA patients independently since it is accurate and stable. Four prognostic ARGs that composed the model can be studied deeply for target treatment.

## 1. Background

Death rates are increasing for oropharyngeal cancer overall by 0.5% per year from 2009 to 2018. There is an estimated number of 54,010 new cases of oropharyngeal cancer (accounting for about 2.8% of new cancers), and 10,850 will die of it (accounting for about 1.8% of all deaths from cancer) in 2021 in the United States [1]. Among all oropharyngeal cancer patients, approximately half of those occur specifically in the oral cavity and were called oral cancer (ORCA) [2]. All over the world, 377,713 people were diagnosed with oral cancer in 2020, and the cancer caused 177,757 deaths; it means there was one person who died due to ORCA every 3 minutes [3]. The treatment and prognosis of ORCA patients relied on the staging system and conventional prognostic factors used in clinical practice, for example, the tumor-node-metastasis (TNM) staging system was the most important and well-known prognostic factor for ORCA patients [4], but the staging system was not accurate enough and prognostic factors were inadequate and nonspecific, leading to discouraging overall prognosis. Recent statistics show that the overall 5-year survival rate for ORCA patients was 52.0% in China [5]. Therefore, searching molecular prognostic markers which could be incorporated into the prognostic system is significant; they can benefit predicting clinical outcomes and targeted therapy. This study attempts to refine the prognostic signature of ORCA and used it in the clinical setting.

Autophagy is a process that can circulation and reuse cellular components; it is nonspecific. Autophagy-related genes (ARGs) are molecules that participate in autophagy. In recent studies, many researchers deemed that autophagy is an important process in ORCA [6–8]. Most of the molecular mechanisms which are involved in autophagy regulation deeply participate in signaling pathways that assumed significant roles in ORCA control [9, 10]. For example, autophagy is a significant AKT/mTOR pathway target, and the antioncogene that negatively regulates mTOR (such as AMPK and PTEN) will activate autophagy. In addition, many autophagy-inducing proteins are either oncoproteins or tumor suppressor proteins [11]. Some studies have proven that the autophagy inhibitor can enhance efficacy against aggressive ORCA [12, 13]. However, how to predict the prognosis of ORCA patients by several ARGs is still unclear. Therefore, our study utilized several screened ARGs to establish the prognostic risk model of ORCA. In this study, the relationship between prognostic ARGs and clinicopathological features in 127 ORCA patients were evaluated, and an autophagy-related signature was constructed to predict survival for ORCA patients. The risk score model had been verified from several aspects, and we proved its accuracy; we also verified the prognosis value of each ARG that comprised the autophagy-related risk model and proved them as prognostic biomarkers in ORCA; our report may shed light on evaluating the prognosis and targeting treatment of ORCA.

## 2. Methods

### 2.1. Data Acquisition

In total, 232 genes presently known as autophagy-related genes (ARGs) were downloaded from HADb (Human Autophagy Database, http://autophagy.lu/). RNA-seq data and clinical features of 127 ORCA patients and 13 nontumor samples were obtained from the TCGA website (The Cancer Genome Atlas database, https://tcga-data.nci.nih.gov/tcga/).

### 2.2. Identification of DEARGs and Functional Annotation

The “limma” package in R was applied to compare differential expressions between ORCA and nontumor counterparts; the criteria is false discovery rate (*FDR*) < 0.05 and ∣log_2_*FoldChange* |  (∣log_2_*FC*∣) > 1. Then, we performed enrichment analyses of Gene Ontology (GO) and Kyoto Encyclopedia of Genes and Genomes (KEGG) for DEARGs to find the major biological attributes. The R packages of ClusterProfiler and org.Hs.eg.db were used to perform GO and KEGG function enrichment analyses. The visualization of results was implemented with the R “ggplot2” package. STRING database (https://string-db.org/) was used to construct the protein–protein interaction (PPI) network of DEARGs. Cytoscape software visualized the interaction of the PPI network.

### 2.3. Construction and Verification of ARG-Based Prognostic Risk Model

Univariate Cox proportional risk regression analysis was performed to screen prognostic ARGs. All statistically significant prognostic ARGs were selected as candidates for multivariate Cox regression analysis and used to construct the prognostic risk model. The multivariate analysis provided the regression coefficient of prognostic ARGs in the risk score model.

(1) $$\begin{array}{c} \text{The risk score}=\underset{i=1,2,\cdots ,n}{\sum }\text{r}\text{egression coefficient}\left(\text{genei}\right)\times \text{expression value of}\left(\text{genei}\right). \end{array}$$

The median risk score was determined as the threshold to divide the ORCA patients into the high- or low-risk group. Four risk genes comprising the risk model were used to construct the nomogram. We constructed a nomogram based on 4 independent prognosis-related ARGs to predict survival rates of patients at 1, 2, and 3 years via the rms package in R 4.0.1.

The predictive accuracy of the risk models was evaluated by the receiver-operator characteristic (ROC) analysis; besides, we compared survival probability of high-risk and low-risk groups using the “ggplot2” package in R. We also assessed the association between the prognostic risk model and classical clinical parameters (such as tumor grade, pathological stage, and T/N classification) by Cox proportional hazard regression analysis.

### 2.4. Validation for Prognostic Value of 4 Risk ARGs which Formed the Model

The relationships between expressions of 4 independent prognostic-related ARGs (*WDR45*, *MAPK9*, *VEGFA*, and *ATIC*) and OS of ORCA patients were evaluated individually by Kaplan–Meier survival curves. We also observed the mRNA expression pattern of the 4 genes in ORCA and normal tongue tissues via the Oncomine database (https://www.oncomine.org/).

To explore the roles of 4 risk ARGs in ORCA, a single-gene gene set enrichment analysis (GSEA) was performed. GSEA was conducted using a molecular signature database's (MSigDB's) c2.cp.kegg.v7.0.symbols.gmt gene sets in GSEA 3.0 software to identify gene sets that were significantly correlated with the expression of risk genes. We exhibited the top 10 biological processes most associated with the expression of the 4 risk genes.

## 3. Results

### 3.1. Identification of DEARGs

**Figure 1:**
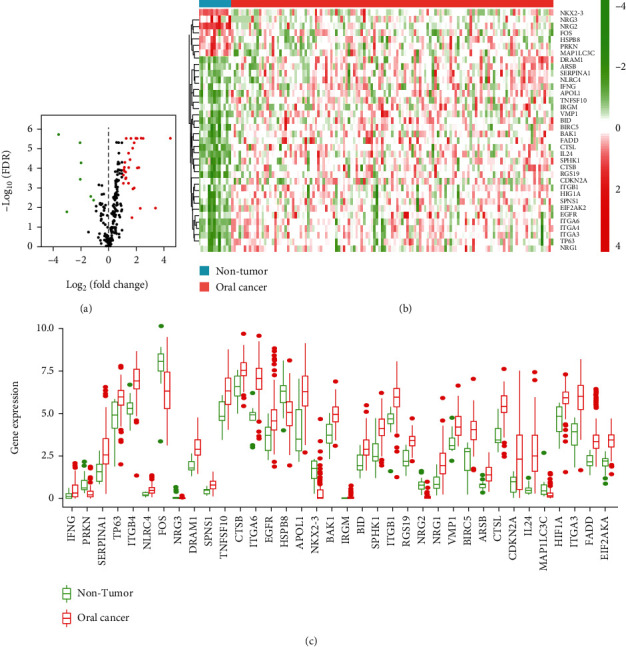
Differentially expressed autophagy-related genes (DEARGs) in ORCA tissues. (a) Volcano plot of autophagy-related genes (ARGs) in ORCA. Green: downregulated genes; red: upregulated genes. (b, c) Heat map and boxplot of the expression levels of 36 DEARGs in ORCA. ORCA: oral cancer.

The expression profile of 232 ARGs in 127 ORCA tissue samples and 13 normal mouth samples were compared and analyzed. Finally, there were 36 differentially expressed ARGs including 29 upregulated ARGs and 7 downregulated ARGs with a threshold of ∣log_2_*FoldChange* | >1. The volcano plot, heat map, and box plot visualized the expression pattern of all DEARGs between ORCA and normal tissues (Figures 1(a)–1(c)). We listed all DEARGs in Table 1, including the log_2_FoldChange and statistical significance.

### 3.2. Functional Annotation for All DEARGs

**Figure 2:**
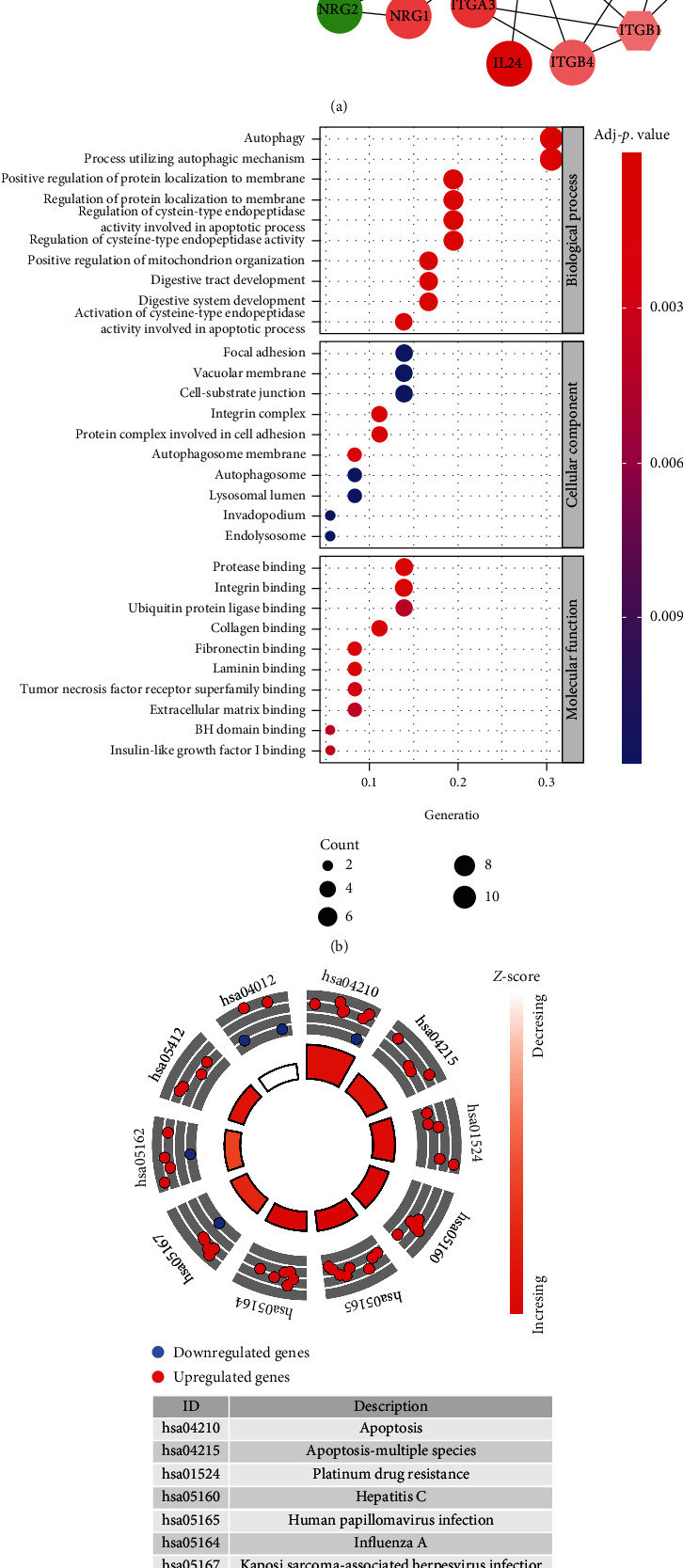
PPI network and functional enrichment analysis for 36 DEARGs. (a) PPI network of 36 DEARGs. Diamond nodes are hub genes which interact with more than 5 genes. Red nodes were upregulated DEARGs; green nodes were downregulated DEARGs. The color depth and size of nodes were related to log_2_FoldChange and *p* value, respectively. The width of links was significantly associated with combined score of protein interaction. (b) Gene Ontology analysis of DEARGs. (c) The circle plot of KEGG pathway analyses for DEARGs.

In Figure2(a), all DEARGs are linked to form a protein-protein interaction network. The DEARGs are enriched in several biological processes (BP) such as autophagy and protein localization. In the molecular function (MF) term of GO analysis, some protein binding-related functions including “protease binding,” “integrin binding,” and “ubiquitin protein ligase binding” were significant roles these DEARGs played. For cellular components (CC), “focal adhesion,” “vacuolar membrane,” and “cell substrate” were significantly enriched (Figure2(b)). KEGG analysis exhibited that the DEARGs were significantly associated with apoptosis, platinum drug resistance, and hepatitis C (Figure2(c)).

### 3.3. Establishment of Prognosis Prediction Model with ARGs

**Figure 3:**
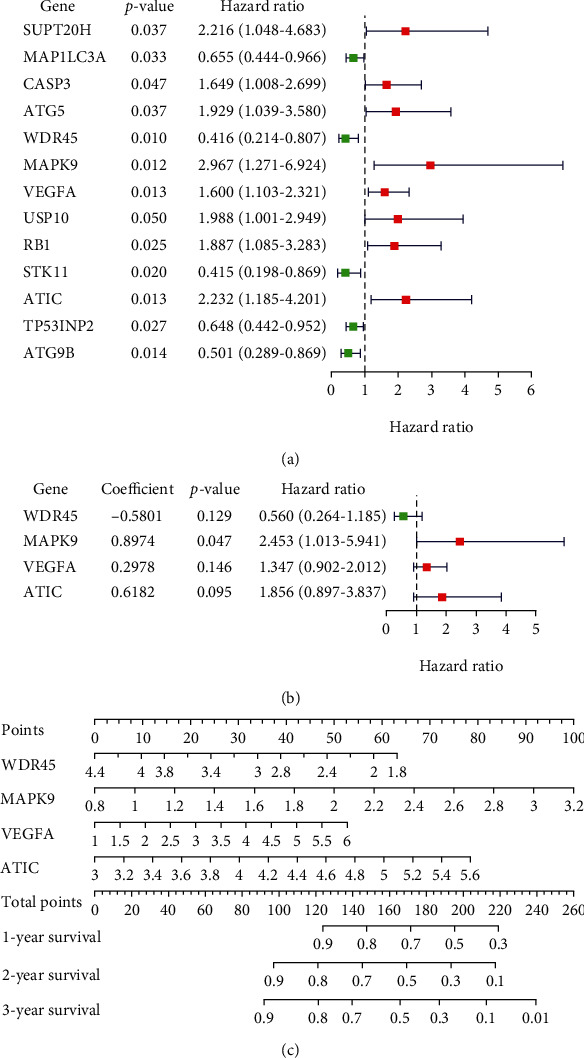
Establishment of a prognostic risk model making use of ARGs for ORCA patients. (a) 13 ARGs were prognostic genes based on univariate Cox analysis. (b) Regression coefficients, hazard ratios, and *p* value of 4 independent prognostic ARGs obtained from multivariate Cox model. (c) The nomogram prediction for overall survival probability at 1, 2, and 3 years for ORCA patients in TCGA cohort.

We performed the univariate Cox proportional hazard analysis on all ARGs; a total of 13 ARGs were significantly associated with the prognosis of ORCA patients (*p* < 0.05) (Figure 3(a)). Then, these prognostic ARGs entered the multivariate Cox regression analysis, and finally, 4 of them (*WDR45*, *MAPK9*, *VEGFA*, and *ATIC*) were screened as independent prognostic genes to construct the prognosis prediction model (Figure 3(b)). For each ORCA patient, the risk score = (−0.5801 × expression value of *WDR*45) + (0.8974 × expression value of *MAPK*9) + (0.2978 × expression value of *VEGFA*) + (0.6182 × expression value of *ATIC*). We divided the 126 ORCA cases into high- or low-risk groups according to the median values of the risk score. In Figure 3(c), the above 4 independent prognostic ARGs were incorporated into a nomogram model for predicting the individualized probability of survival times in clinical practice in ORCA patients. The score of every ARG can be found through the point scale located at the top of the nomogram. Then, the points of each ARG were summed, thereby estimating survival probability at 1, 2, and 3 years.

### 3.4. Testing the Risk Model

**Figure 4:**
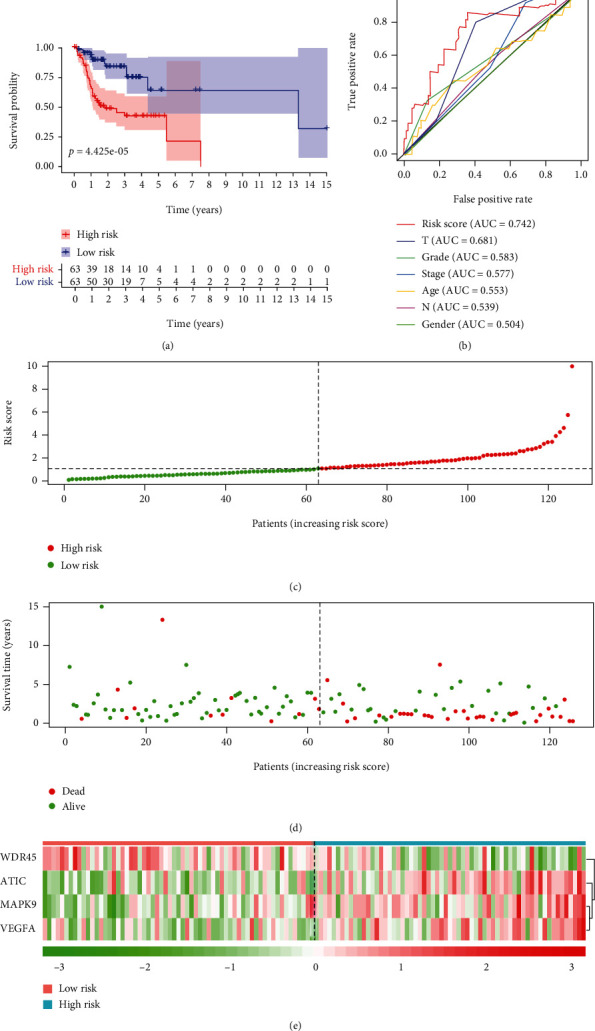
Validation of the prognostic risk model. (a) Kaplan-Meier curves of high-risk and low-risk ORCA patients. (b) ROC curves of risk score and traditional clinical parameters for prognosis predicting. (c) The distribution of risk scores. Each dot represents an ORCA patient. (d) The survival status of ORCA patients with different risk scores. (e) Heat map of 4 prognostic ARGs revealed the relationship between risk score and risk gene expression level.

Patients with ORCA were stratified into high- and low-risk groups based on the median risk score, and Kaplan-Meier plots indicated that patients in the low-risk group had a significantly better prognosis (*p* = 4.425*E* − 05) (Figure 4(a)). The results of ROC curves revealed that this prognostic risk model had good predictive performance in predicting the overall survival of ORCA patients with areas under the ROC curve (AUCs) of 0.742 (Figure 4(b)). Figures 4(c)–4(e) indicate the risk distribution of patients, survival time of patients in high- and low-risk groups, and expression profile heat map of the 4 ARGs that formed the risk model.

**Figure 5:**
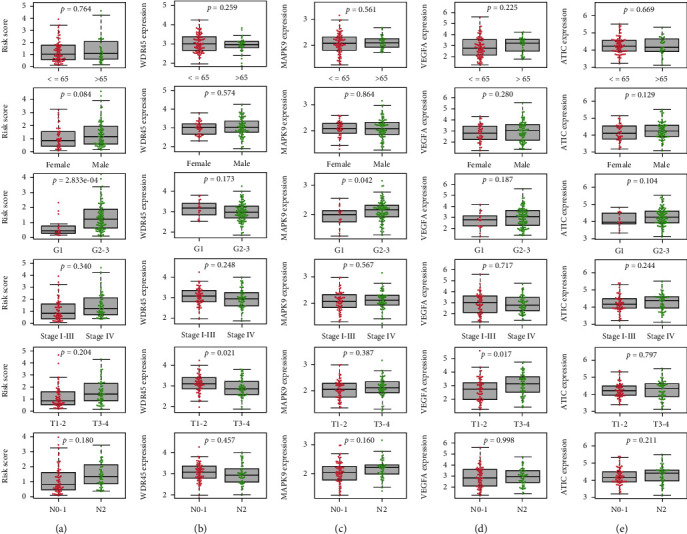
Clinical correlations between traditional clinicopathological indicators (age, gender, grade, pathological stage, T classification, and N classification) and the risk score (a). The association between expression of 4 prognostic ARGs [(b) *WDR45*, (c) *MAPK9*, (d) *VEGFA*, and (e) *ATIC*] and clinicopathological features.

We grouped the patients by age, sex, histological grade, clinical stage, and T/N classification to explore the association between the risk model and clinicopathological features. In Figure 5, we can find a trend of risk scores which were higher in patients classified with high grade, advanced pathologic stage, and terminal T/N stage (Figure 5(a)). The expressions of *MAPK9*, *VEGFA*, and *ATIC* all had a trend of being upregulated in patients with high grade, advanced pathologic stage, and terminal T/N stage; in addition, the expressions of *WDR45* had an opposite trend (Figures 5(b)–5(e)). The expression regularity of 4 independent prognostic ARGs that comprised the risk model in patients with different clinicopathological features was in accordance with their expression trend in different risk groups. The expressions of *MAPK9*, *VEGFA*, and *ATIC* were higher in the high-risk group, and the expressions of *WDR45* was higher in the low-risk group (Figure 4(e)). It proved that the risk prognostic model was better than any other clinicopathological features for predicting the prognosis for ORCA patients.

The results in Table 2 suggested that our prognostic risk model could independently predict the prognosis of ORCA. Although T classification was also significantly associated with survival, ROC analysis demonstrated that T classification (*AUC* = 0.681) was not as reliable as the risk score (*AUC* = 0.742).

### 3.5. Validation of 4 Risk Genes that Comprised the Risk Model

**Figure 6:**
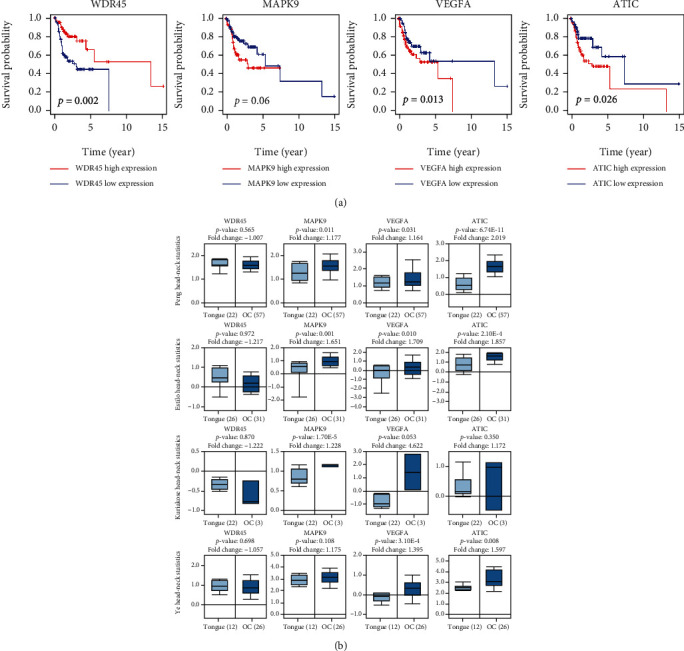
The correlation between 4 risk ARGs (*WDR45*, *MAPK9*, *VEGFA*, and *ATIC*) and overall survival of ORCA patients (a). The expressions of 4 risk genes (*WDR45*, *MAPK9*, *VEGFA*, and *ATIC*) between normal mouth and ORCA tissues were compared in Oncomine database (b).

**Figure 7:**
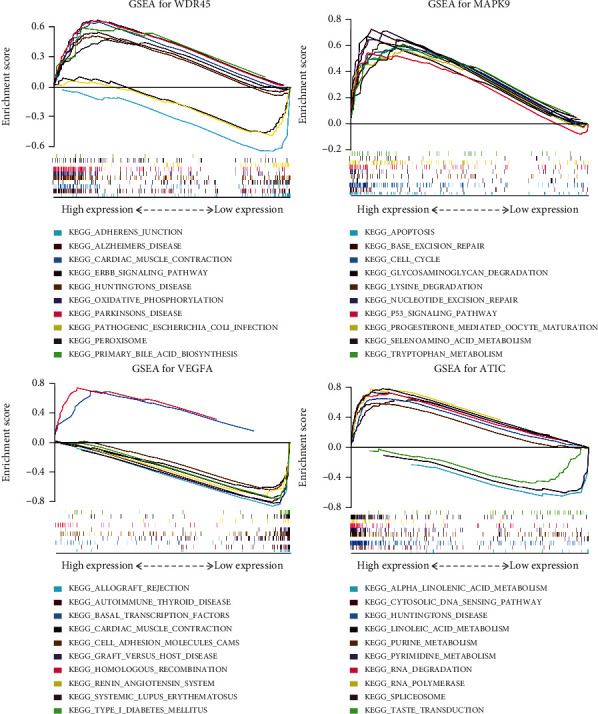
Single-gene GSEA analysis of 4 prognostic ARGs which formed the prognostic risk model in ORCA.

*WDR45*, *MAPK9*, *VEGFA*, and *ATIC* were 4 prognostic-related ARGs which formed the prognostic risk model. As shown in Figure 6(a), upregulation of *WDR45* was strongly correlated with longer patient survival, and *MAPK9*, *VEGFA*, and *ATIC* overexpression reduced OS in patients with ORCA. We also verified the expression of 4 risk genes in the Oncomine database (Figure 6(b)) and found that the expression trend of 4 prognostic ARGs is in accordance with their expression patterns in ORCA, and there are no tumor tissues in previous results in this paper in the TCGA database as shown in Figure 4(e) and Figures 5(b)–5(e). Single-gene GSEA of the 4 risk genes revealed their potential function in ORCA (Figure 7).

## 4. Discussion

Autophagy is a conserved and dynamic process whose function maintains cellular homeostasis [14]. Multiple studies had confirmed that autophagy played a significant role in many cancers [15–17]. Important experimental evidences had proven autophagy's potential as a therapeutic target for ORCA [18–20]. Hence, we constructed an autophagy-related prognostic risk model in this paper for prognosis prediction for ORCA patients.

The autophagy-related prognostic model is formed by 4 ARGs, including *WDR45*, *MAPK9*, *VEGFA*, and *ATIC*. According to our risk model, the expressions of *MAPK9*, *VEGFA*, and *ATIC* in the high-risk group were higher than those in the low risk-group; it is worth noting that their expressions in ORCA tissues were higher than those in the control, and their expressions all had a trend of upregulation in patients with advanced pathologic stage and high grade; in addition, high expressions of the 3 genes were significantly related to worse OS. What this means is that *MAPK9*, *VEGFA*, and *ATIC* were indeed high-risk factors in ORCA. The expression law of *WDR45* was opposite to *MAPK9*, *VEGFA*, and *ATIC* and proved it was a low-risk factor in ORCA. The risk score model is significantly associated with clinicopathological indicators of ORCA patients. Our paper proved that the risk model comprised of *WDR45*, *MAPK9*, *VEGFA*, and *ATIC* for evaluating the prognosis of ORCA patients is clinically practicable.

*MAPK9* is also called *JNK2*; recent studies have highlighted the oncogenic potential of *MAPK9* in several human cancer cells, such as lung [21] and glioblastoma [22]. The role of *MAPK9* in oral cancer is still controversial [23]. *VEGFA* encoded vascular endothelial growth factor A; many researchers reported that it was upregulated in oral squamous cell carcinomas [24, 25]. *ATIC* was considered an effective target for chemoradiosensitization [26]. Although there are some researches about independent prognostic ARGs in recent years, they are not incorporated into a prognostic evaluation system in the right proportions and ways, so exploring an effective prognostic risk model and novel targets for ORCA therapy is necessary.

Functional enrichment analysis showed that 36 DEARGs were mainly involved in apoptosis and inflammation-associated pathways. Dwivedi et al. reported that to regulate apoptosis may be the best way against ORCA [27]. A lot of reports deemed that oral inflammation promotes ORCA [28, 29]. Hence, clinical doctors maybe should pay more attention to the effect of inflammation on ORCA patients.

In this paper, we demonstrated that the novel prognostic risk model had excellent potential as a prognostic predictor which is even better than traditional clinicopathological indicators in ORCA. It can be incorporated into the clinical evaluation indicators to better predict clinical outcomes. Individual assessment of 4 risk genes that comprised the risk model in ORCA further proved that *WDR45*, *MAPK9*, *VEGFA*, and *ATIC* all play important parts in ORCA.

## 5. Conclusions

A novel autophagy-related model based on the expression levels of 4 risk genes was explored for survival prediction for ORCA patients. The 4 risk genes can benefit the underlying molecular mechanisms of ORCA and be utilized as potential therapeutic targets.

## Figures and Tables

**Table 1:** All DEARGs, screened between normal mouth tissues and oral cancer tissues with criteria of FDR < 0.05 and ∣log_2_FoldChange | >1.

Gene	Log_2_FC	*p* value	FDR
IFNG	1.69529	0.02038	0.03289
PRKN	-1.97667	5.69*E*-06	5.30*E*-05
SERPINA1	1.87471	0.00030	0.00100
TP63	1.07599	0.00015	0.00058
ITGB4	1.73712	6.51*E*-07	9.53*E*-06
NLRC4	1.25024	6.76*E*-05	0.00031
FOS	-1.27976	0.00098	0.00276
NRG3	-3.00689	0.00871	0.01666
DRAM1	1.46342	1.35*E*-06	1.69*E*-05
SPNS1	1.13948	1.15*E*-05	9.07*E*-05
TNFSF10	1.44512	0.00015	0.00059
CTSB	1.04712	4.40*E*-05	0.00021
ITGA6	2.50098	1.04*E*-07	2.99*E*-06
EGFR	1.76767	0.00033	0.00109
HSPB8	-1.07785	0.00166	0.00420
APOL1	2.08150	1.27*E*-05	9.30*E*-05
NKX2-3	-2.06465	3.12*E*-07	4.92*E*-06
BAK1	1.13452	2.07*E*-05	0.00013
IRGM	2.29365	0.00536	0.01098
BID	1.23911	2.67*E*-05	0.00015
SPHK1	1.54880	3.17*E*-06	3.61*E*-05
ITGB1	1.46611	1.11*E*-05	9.07*E*-05
RGS19	1.27753	9.97*E*-08	2.99*E*-06
NRG2	-3.60739	9.26*E*-09	1.90*E*-06
NRG1	2.20708	1.23*E*-05	9.30*E*-05
VMP1	1.13398	1.88*E*-05	0.00012
BIRC5	2.00367	1.17*E*-07	2.99*E*-06
ARSB	1.22169	7.62*E*-05	0.00035
CTSL	1.85491	2.84*E*-07	4.92*E*-06
CDKN2A	3.39449	0.00511	0.01069
IL24	4.48142	7.85*E*-08	2.99*E*-06
MAP1LC3C	-2.03068	8.33*E*-05	0.00037
HIF1A	1.17363	4.80*E*-06	5.01*E*-05
ITGA3	2.34824	1.04*E*-07	2.99*E*-06
FADD	2.08295	1.12*E*-07	2.99*E*-06
EIF2AK2	1.61684	6.43*E*-08	2.99*E*-06

**Table 2:** Univariate and multivariate Cox regression analyses of risk score and clinicopathologic features in the TCGA group oral cancer patients.

Variables	Univariate analysis		Multivariate analysis	
	HR (95% CI)	*p* value	HR (95% CI)	*p* value
Risk score	1.622(1.354−1.942)	**<**0.001	1.628(1.327−1.997)	**<**0.001
Age	1.006(0.979−1.035)	0.647	1.002(0.967−1.038)	0.916
Gender	0.798(0.408−1.561)	0.510	0.604(0.300−1.215)	0.157
Grade	2.043(1.146−3.643)	0.015	1.510(0.770−2.960)	0.230
Pathologic stage	1.547(1.047−2.285)	0.028	0.851(0.356−2.031)	0.716
T classification	1.724(1.214−2.447)	0.002	1.715(1.014−2.902)	0.044
N classification	1.481(1.023−2.143)	0.037	1.356(0.712−2.583)	0.355

## Data Availability

The raw data supporting the conclusions of this manuscript will be made available by the authors, without undue reservation, to any qualified researcher.

## Abbreviations

ARGs: Autophagy- related genes
AUC: Area under the curve
DEARGs: Differentially expressed autophagy- related genes
DEGs: Differentially expressed genes
FDR: False discovery rate
GO: Gene Ontology
GSEA: Gene set enrichment analysis
KEGG: Kyoto Encyclopedia of Genes and Genomes
KM plotter: Kaplan-Meier plotter
Log_2_FC: Log_2_FoldChange/logarithm of fold change
ORCA: Oral cancer
OS: Overall survival
PPI: Protein-protein interaction
ROC: Receiver-operator characteristic
TCGA: The Cancer Genome Atlas.
